# Interaction of *CPR5* with Cell Cycle Regulators *UVI4* and *OSD1* in Arabidopsis

**DOI:** 10.1371/journal.pone.0100347

**Published:** 2014-06-19

**Authors:** Zhilong Bao, Jian Hua

**Affiliations:** Department of Plant Biology, Cornell University, Ithaca, New York, United States of America; UMBC, United States of America

## Abstract

The impact of cell cycle on plant immunity was indicated by the enhancement of disease resistance with overexpressing *OSD1* and *UVI4* genes that are negative regulators of cell cycle controller APC (anaphase promoting complex). *CPR5* is another gene that is implicated in cell cycle regulation and plant immunity, but its mode of action is not known. Here we report the analysis of genetic requirement for the function of *UVI4* and *OSD1* in cell cycle progression control and in particular the involvement of *CPR5* in this regulation. We show that the APC activator CCS52A1 partially mediates the function of *OSD1* and *UVI4* in female gametophyte development. We found that the *cpr5* mutation suppresses the endoreduplication defect in the *uvi4* single mutant and partially rescued the gametophyte development defect in the *osd1 uvi4* double mutant while the *uvi4* mutation enhances the *cpr5* defects in trichome branching and plant disease resistance. In addition, cyclin B1 genes *CYCB1;1*, *CYCB1;2*, and *CYCB1;4* are upregulated in *cpr5*. Therefore, *CPR5* has a large role in cell cycle regulation and this role has a complex interaction with that of *UVI4* and *OSD1*. This study further indicates an intrinsic link between plant defense responses and cell cycle progression.

## Introduction

Regulation of cell cycle, in the form of meiosis, mitosis, and endoreduplication, is critical for plant growth and development [1]–[4]. Progression through cell division cycles is governed by activities of cyclin-dependent kinase (CDK)-cyclin complexes which are bound and activated by cyclins at specific cell cycle phases. Eight classes of CDKs including CDKA to CDKG and CDK-like kinases (CKLs) were identified in Arabidopsis, but only CDKA and CDKB were reported to be involved in the regulation of cell cycle progression [5]. During mitotic cell cycles, activities of CDKs are relatively high at G1/S and G2/M transition phases where a large number of proteins are phosphorylated to promote the onset of DNA replication and mitosis respectively. During endocycles, the activity of mitotic CDK-cyclin complex at the G2/M transition phase needs to be repressed, which could be achieved by activation of the anaphase-promoting complex/cyclosome (APC/C).

APC/C is a multi-subunit E3 ubiquitin-ligase that degrades cell cycle proteins to control cell cycle progression [6], [7]. The activity and substrate specificity of APC/C activity is controlled by two types of activators: *Cell division cycle 20/Fizzy* (*CDC20/FZY*) and *CDC20 homolog/Fizzy-related* (*CDH1/FZR*). Mitotic cell cycle progression requires the function of both *CDC20* and *CDH1*, while the onset and progression of endocycles are controlled by *CDH1* only. Arabidopsis has five *CDC20* homologs (*CDC20.1* to *CDC20.5*) and three *CDH1* homologs (*CCS52A1*, *CCS52A2*, and *CCS52B*). Both *CCS52A1* and *CCS52A2* are reported to regulate the onset of endoreduplication, but the function of *CCS52B* is largely unknown [8]–[11].

Two homologous genes *OSD1/GIG1* (*Omission of the Second Division/gigas cell 1*) and its homolog *UVI4* (*UV Insensitive 4*) are negative regulators of APC/C [12], [13]. The loss of *OSD1* function led to omission of the second meiotic division and a subsequent production of diploid gametes [14]. A double mutant of *cyca1;2* and *osd1* had no chromosome segregation during male meiosis, indicating that *OSD1* promotes transitions in both meiotic divisions [15]. The mutant *osd1/gig1* has gigantic cotyledon epidermal cells with higher ploidy, indicating a role of *OSD1* in endoreduplication or endomitosis in cotyledons [13]. The loss of *UVI4* function leads to enhanced resistance to UV-B and increased ploidy level in somatic tissues, indicating that *UVI4* inhibits endocycles [16], [17]. Interactomics experiments by overexpressing core cell cycle genes in Arabidopsis suspension cell culture revealed that both OSD1 and UVI4 interact with the APC/C complex [18]. Yeast two-hybrid analyses supported an interaction of both UVI4 and OSD1 with the catalytic activator subunits of APC/C including CCS52A1, CCS52B, CDC20.1, and CDC20.5 [12], [13]. It is likely that more than one of these activators mediate the function of OSD1 or UVI4. While the *ccs52a1* mutation largely suppressed the enhanced endoreduplication defect in *uvi4* [12], overexpression of *CDC20.1* or *CCS52B* was reported to further enhance the ploidy level in *osd1* and *uvi4* mutants [13]. Multiple cyclin proteins are regulated by *OSD1* and *UVI4*. Increased degradation of the CYCA2;3 protein was observed in the *uvi4* mutant while transient overexpression of *UVI4* or *OSD1* under the dexamethasone-inducible promoter triggered higher accumulation of CYCB1;2 and CYCB1;1 proteins [12], [13], [19].

Intriguingly, perturbation of cell cycle progression affects plant immune responses. Overexpression of either *UVI4* or *OSD1* and reduction of the function of *APC10* resulted in enhanced disease resistance against virulent bacterial pathogen via upregulation of disease resistance (*R*) genes in a *CYCB1;1-*dependent manner [20]. *R* genes encode plant immune receptors that recognize directly or indirectly effector proteins secreted from pathogens, and activation of R proteins leads to transcriptional reprogramming and often programmed cell death to inhibit the spreading of pathogens in plants. There is therefore an apparent connection between cell cycle progression and disease resistance that is often associated cell death in plants. In animal and fungal systems, cell cycle progression is tightly linked to cell survival. Cell damage is assessed by various cell cycle checkpoints and either causes cell cycle arrest for DNA repair or leads to cell death [21]. In plants, a few examples exist for the association of cell cycle arrest and disease resistance associated with cell death. In addition to *OSD1* and *UVI4* that affect both cell cycle and disease resistance, the Arabidopsis *CPR5* gene is implicated in both processes. The loss-of-function (l-o-f) *cpr5* mutant shows increased disease resistance to bacterial pathogens accompanied by high accumulation of salicylic acid and ectopic cell death [22], [23]. It also has abnormal trichomes due to reduced endoreduplication and cell death [24]. The *cpr5* mutant has additional mutant phenotypes including early senescence, hyper sensitivity to sucrose [25], low leaf potassium content [26], abnormal response to ABA [27], and abnormal cell wall biosynthesis [28]. *CPR5* is likely a component of a general biochemical or cellular process and thus has a broad impact on different processes. Little is known about the biochemical properties of CPR5 besides that it has a transmembrane segment and is localized to both cytoplasm and nucleus [29].

Here we report the analyses of genetic interactions between *OSD1*, *UVI4*, *CCS52A1* and *CPR5* genes in the regulation of cell cycle progression. Loss of function mutations of *osd1* and *uvi4* individually promote endoreduplication and together lead to lethality of female gamete. We show that the lethality of *osd1 uvi4* could be partially suppressed by a mutation in the APC activator *CCS52a1*. Interestingly, the *cpr5* mutation suppressed many defects of *uvi4* single mutant and the lethality of the *osd1 uvi4* double mutant while the *uvi4* mutation enhanced the *cpr5* defect in trichome branching and disease resistance. In addition, the expression of *CYCB1;1* and *CYCB1;2* genes are upregulated in *cpr5*. These data indicate that *CPR5* has a critical role in cell cycle regulation and this role has a complex interaction with those of *OSD1* and *UVI4*. It further indicates an intrinsic connection between cell cycle regulation and plant immunity.

## Materials and Methods

### Plant Materials, Growth and Transformation

Seeds of SALK_083656 were obtained from Arabidopsis Biological Research Center (ABRC). Heterozygous Seeds of *osd1-2* (GT21481) were obtained from cold spring harbor laboratory. The *osd1-2^C^* mutant was introgressed from heterozygous *osd1-2* into Col-0 for seven times. Plants were grown under either 12 hour light/12 hour dark or constant light condition at 22°C. Plant transformation was performed as previously described [30]–[32].

### Bacterial Growth Assay

Four-week old plants grown under 12 hour light/12 hour dark condition were inoculated by *Pst* DC3000 at the concentration of 1×10^8^ colony forming units (cfu)/ml (OD600 = 0.2), and bacterial growth in different genotypes was analyzed at day 3 after the inoculation [33].

### Ploidy Measurement

The first and second true leaves from two plants of 4-week old were collected and chopped in “Aru” buffer containing 97.5% MgSO_4_ (0.246% MgSO_4_^.^7H_2_O, 0.37% KCl and 0.12% Hepes), 0.1% DTT and 2.5% Triton X-100 [34]. 10 µl of PI (propidium iodide) stock solution (5 mg/ml) and 5 µl RNase stock solution (10 mg/ml) were added into each sample of approximately 600 µl. Beckman-Coulter Epics XL-MCL flow cytometer was used to measure ploidy with rice and maize leaf samples as controls. Three replicas were analyzed for each sample. Ploidy index (PI) was calculated by the formula: PI =  (%2C nuclei×1) + (%4C nuclei×2) + (%8C nuclei×3) + (%16C nuclei×4) + (%32C nuclei×5). Independent experiments were conducted at least twice. The representative data were shown in figures.

### Confocal Microscopy

The development of ovules was analyzed by using Leica TCS SP2 confocal microscope according to protocols previously described [35], [36].

### Quantitative RT-PCR

qRT-PCR was conducted by using FastStart universal SYBR Green Master mix (Roche). All primers are listed in Table S1 [37].

## Results

### Overexpression of *OSD1* and *UVI4* Affects Endoreduplication in Leaves

**Figure 1:**
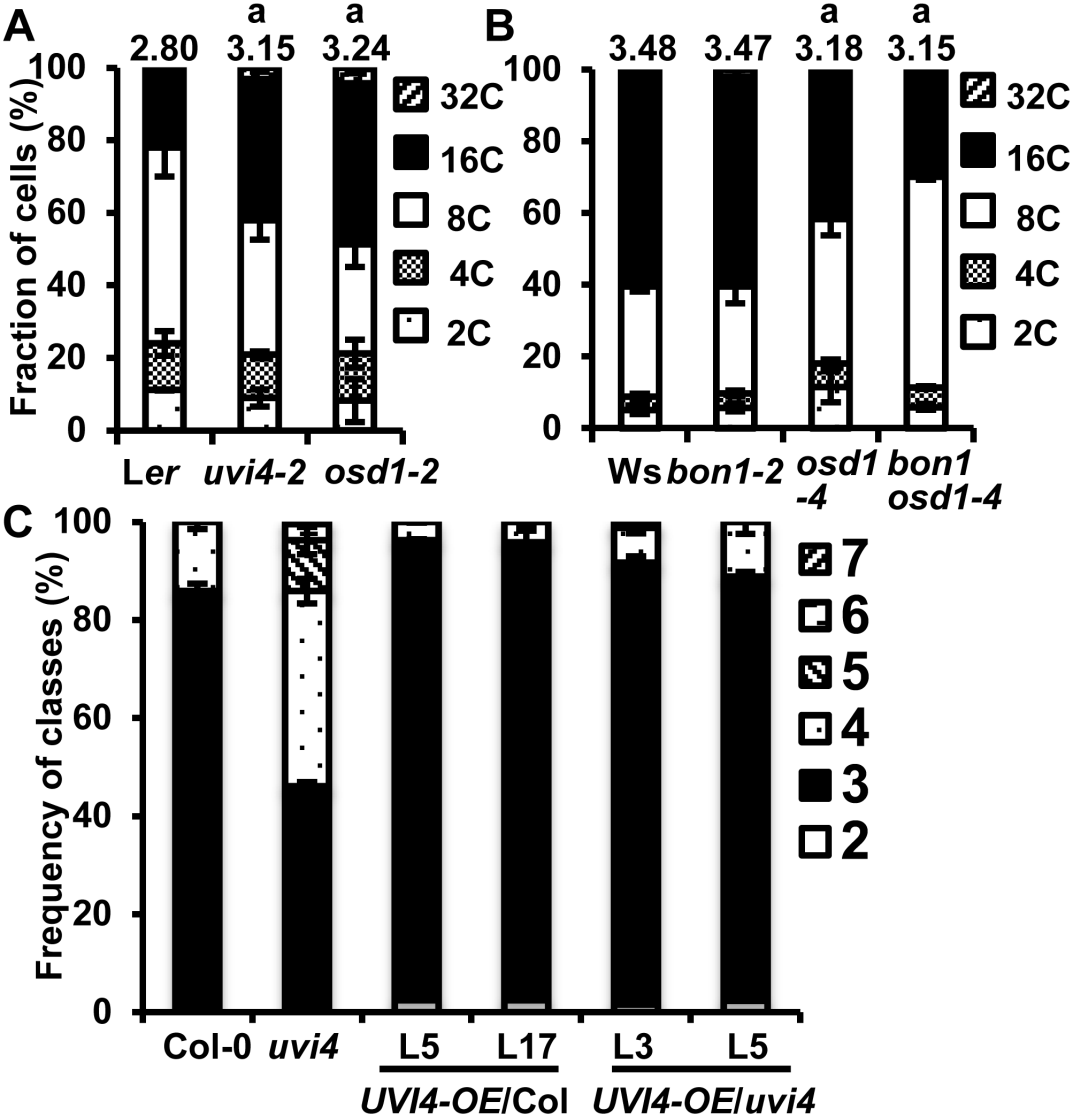
Overexpression of *OSD1* and *UVI4* affect endoreduplication in leaves. (A) Ploidy levels in the first pair of leaves from the wild-type L*er*, *uvi4-2*, and *osd1-2* grown under 12 hour light/day for 4 weeks. (B) Ploidy levels in the first pair of leaves from Ws, *bon1-2*, *osd1-4* and *bon1 osd1-4* grown under 12 hour light/day for 4 weeks. Error bars indicate standard deviations. The number above the column indicates the ploidy index. Averages of three replicas for each sample were shown in (A) and (B). Ploidy indices were used for statistical analysis, and the letter a indicates a statistical significance determined by student *t*-test. The representative data were shown from two independent measurements. (C) Frequencies of each class of trichome numbers in Col-0, *uvi4* and *UVI4-OE* transgenic lines 5 and 17 in Col-0 and lines 3 and 5 in *uvi4*. Approximately 100 to 150 cells were examined for each genotype. Error bars indicate standard deviations. The difference between overexpression lines and wild-type Col-0 or *uvi4*was determined by chi-square test (*UVI4-OE/*Col-0 compared to Col-0, L5: P = 0.01<0.05, L17: P = 0.00<0.05; *UVI4-OE/uvi4* compared to Col-0, L3: P = 0.02<0.05, L5: P = 0.21>0.05; *UVI4-OE/uvi4* compared to *uvi4*, L3: P = 0.00<0.05, L5: P = 0.00<0.05).

*OSD1* has an essential role of cell cycle regulation in gametophyte development and cotyledon development. To determine whether or not *OSD1* also has a role in vegetative growth, we analyzed ploidy levels of leaf cells in both loss of function mutants and overexpression transgenic lines of *OSD1*. Because homozygous *osd1-2* plants produce diploid male and female gametes, and thus tetraploid progenies, we selected homozygous *osd1-2* plants (a transposon mutant GT21481) in the Landsberg *erecta* (L*er*) background for ploidy analysis from progenies of heterozygous rather than homozygous *osd1-2* plants. The control was an *uvi4-2* allele in L*er* (Landsberg *erecta*) previously named *pym* [17]. In the first pair of leaves of 4-week old seedlings, *uvi4-2* had an increase of higher-ploidy cells (32C and 16C) as analyzed by flow cytometry (Figure 1A), which is consistent with the previous finding for the *uvi4-1* mutant in Col-0. The *osd1-2* mutant in L*er* also had a significant increase of the number of 16C and 32C cells compared to wild-type L*er* (Figure 1A). Ploidy indices were calculated as 2.80 in L*er*-0, 3.15 in *uvi4-2* and 3.24 in *osd1-2* (Figure 1A). Both *uvi4-2* and *osd1-2* mutants had significantly higher ploidy indices than L*er*-0. Therefore, *OSD1* also has a role in the development of true leaves by inhibiting endoreduplication.

The effect of *OSD1* overexpression on cell cycle progression was examined in the *OSD1* overexpression allele *osd1-4* resulting from activation tagging mutagenesis [20]. This *osd1-4* mutation enhanced defense responses of *bon1-*2, a l-o-f mutant of an immunity negative regulator *BONZAI1* (*BON1*) in the Ws ecotype [20]. A reduced ploidy level in leaf cells was observed in the *osd1-4* mutant compared to the wild type, especially when plants were grown under weaker light illumination (Figure 1B). The *osd1-4* plant had more cells with 2C, 4C, and 8C at the expense of cells with 16C and 32C compared to the wild-type Ws plant (Figure 1B). The *bon1-2* mutation did not alter ploidy distribution in leaf cells, and the *bon1-2 osd1-4* mutant had reduced endoreduplication similarly to *osd1-4*. Ploidy indices were calculated as 3.48 in Ws, 3.47 in *bon1-2*, 3.18 in *osd1-4* and 3.15 in *bon1-2 osd1-4* (Figure 1B). Both *osd1-4* and *bon1-2 osd1-4* had significantly lower ploidy index than Ws and *bon1-2* while no significant difference was observed between *osd1-4* and *bon1-2 osd1-4*, indicating that overexpression of *OSD1* triggers the reduction of endoreduplication. Thus, the level of *OSD1* is critical in controlling ploidy levels in leaf cells, most likely through regulating endoreduplication.

*UVI4* regulates endoreduplication and the loss of *UVI4* function results in higher ploidy level in leaf cells [16]. We tested whether or not a higher expression of *UVI4* can affect cell cycle progression. Overexpression of a UVI4 and GFP fusion as in *UVI4-OE* transgenic plants confers a dwarf phenotype with multiple shoots in both the wild-type Col-0 and the *uvi4-1* mutant in Col-0 (referred to as *uvi4*) [20]. Due to the low germination efficiency, the *UVI4-OE* plants were grown on ½ MS medium for one and half week before being transferred to soil and grown under 12 h light/12 h dark condition for 2 weeks. The fourth leaves of the overexpression lines were used to analyze the trichome phenotype. *UVI4-OE* generated in either Col-0 and *uvi4* had decreased branching trichome number compared to Col-0 or *uvi4* (Figure 1C). While trichome branch numbers in wild-type Col-0 and *uvi4* are more than two, around 2.5% of the trichomes in lines 5 and 17 of *UVI4-OE* in Col-0, and 1.5% and 2.4% of the trichomes in lines 3 and 5 of *UVI4-OE* in *uvi4*, respectively, had only two branches (Figure 1C). Statistical analysis by chi-square test indicates that the distribution of trichome branch numbers in lines 5 and 17 in Col-0 are significantly reduced compared to that in wild type Col-0. Lines 3 and 5 of *UVI4-OE* in *uvi4* had significantly reduced trichome branching numbers compared to *uvi4* but were similar to the wild-type Col-0. These data indicate that overexpression of *UVI4* reduces the level of cell ploidy and the expression level of *UVI4*, like that of *OSD1*, regulates endoreduplication.

### The *ccs52a1* Mutation Leads to a Partial Suppression of the Lethality of *osd1 uvi4*

**Figure 2:**
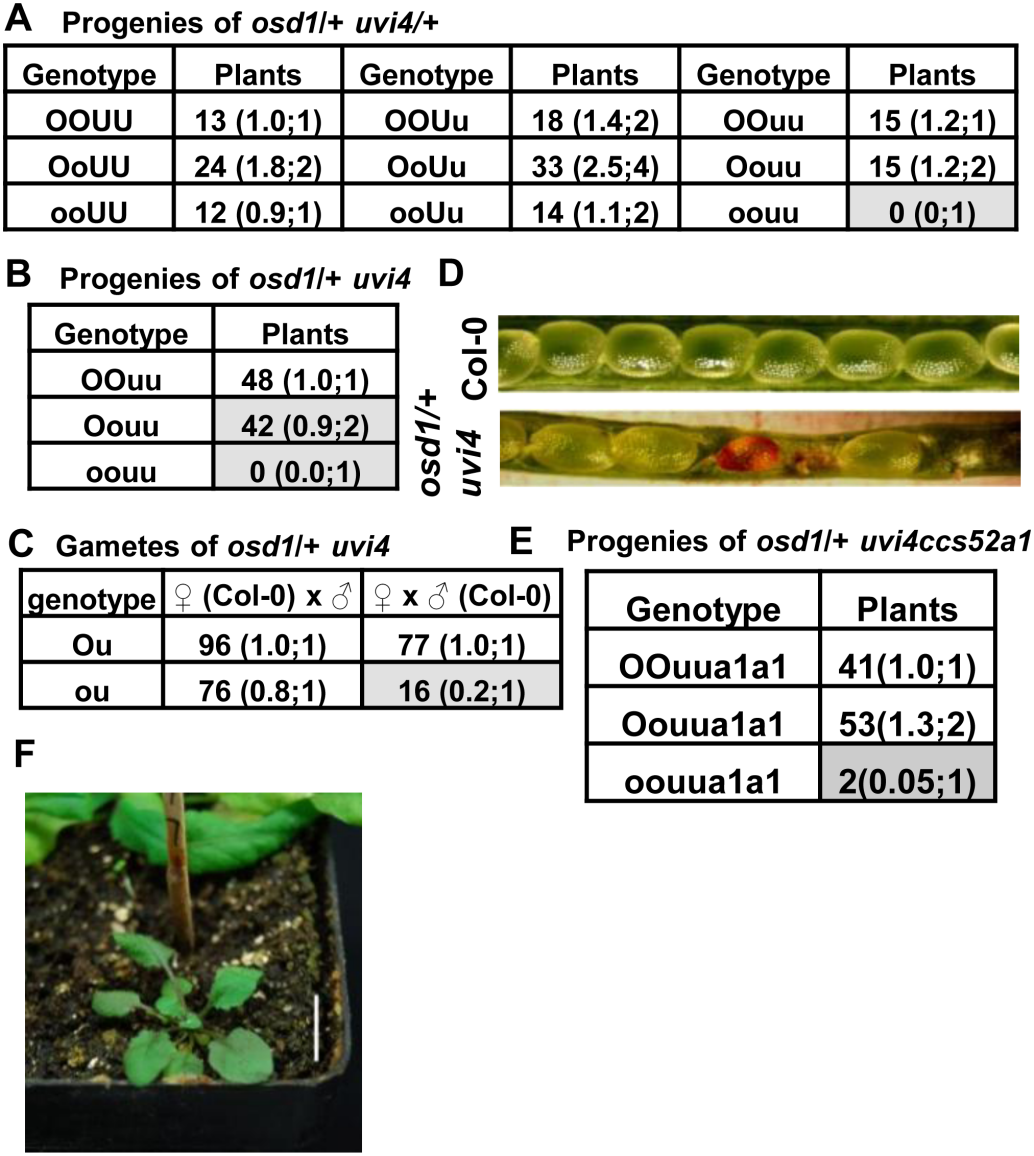
*CCS52A1* partially mediates the lethality of *osd1 uvi4*. Shown are numbers of plants of each genotype in an analyzed population. The two numbers separated by a semicolon in parentheses are the observed ratios of that genotype relative to the top left genotype (left) and the expected ratios when there is no reduced transmission of the gametes or zygotes (right). (A) Analysis of progenies of *osd1/OSD1 UVI4/uvi4*. No *osd1/osd1 uvi4/uvi4* plants (shaded) were found. The difference between observed and expected segregation ratio was determined by chi-square test (P = 0.004<0.05). (B) Analysis of progenies of *osd1*/*OSD1 uvi4/uvi4*. Notice that both *OSD1/OSD1 uvi4/uvi4* and *osd1/OSD1 uvi4/uvi4* (shaded) were about half of expected and there were no *osd1/osd1uvi4/uvi4* (shaded) progenies produced. The significance was determined by chi-square test (P = 6.24E-12<0.05). (C) Analysis of gamete transmission inferred from reciprocal crosses between *osd1/OSD1 uvi4/uvi4* and Col-0. The *osd1 uvi4* genotype had a lower transmission rate through female gametes (shaded) but not male gametes. The significance was determined by chi-square test (male, P = 0.13>0.05; female, P = 2.53E-10<0.05). (D) Opened siliques from wild-type Col-0 (upper) and *osd1/OSD1 uvi4/uvi4* (lower) plants. Aborted ovules and embryos can be seen in the mutant silique. (E) Analysis of progenies of *osd1/OSD1 uvi4/uvi4 ccs52a1/ccs52a1*. Notice that two *osd1/osd1 uvi4/uvi4 ccs52a1/ccs52a1* plants (shaded) were found. (F) Morphology of the *osd1/osd1 uvi4/uvi4 ccs52a1/ccs52a1* triple mutant plant.

We constructed double mutant of *osd1* and *uvi4* and found that it is lethal during both female gametogenesis and embryogenesis (Figure 2A–D, S1), consistent with previous findings [13], [38]. The *osd1-2^C^* mutant was introgressed from *osd1-2* in L*er* to Col-0 using heterozygous mutants for seven times and the resulting mutant in Col-0 is refered as *osd1*. The *osd1* (in a heterozygous state) mutant was crossed to the *uvi4* mutant which is also in the Col-0 background. No homozygous *osd1 uvi4* mutant seedlings could be identified from progenies from a double heterozygous plant *osd1/OSD1 uvi4/UVI4* (Figure 2A) or from *osd1/OSD1 uvi4/uvi4* plants (Figure 2B). Progeny testing from reciprocal crosses between *osd1/OSD1 uvi4/uvi4* and wild-type Col-0 reveals that the transmission rate of the *osd1 uvi4* female gametes was approximately 20% (17/83) of that of the *OSD1 uvi4* female gametes while the that of the *osd1 uvi4* male gametes was not drastically reduced relative to that of the *OSD1 uvi4* male gametes (Figure 2C). Similar to previous report [38], we found that the female gametophyte development in *osd1 uvi4* is arrested at FG1 stage and the megaspore cannot complete mitosis to develop into a functional female gametophyte (Figure S1). Even with some transmission of the *osd1 uvi4* female gametes, the *osd1/osd1 uvi4/uvi4* zygote could not be found in progenies of *osd1/OSD1 uvi4/UVI4* or *osd1/OSD1 uvi4/uvi4* (Figure 2A, B), indicating the *OSD1* and *UVI4* together are essential for zygote development as well. Indeed, undeveloped ovules and arrested embryos were observed in siliques of the *osd1/OSD1 uvi4/uvi4* plant (Figure 2D).

In contrast to *OSD1* and *UVI4*, *CCS52A1* positively regulates the activity of APC/C [10], and *ccs52a1* suppressed the endoreduplication phenotype of *uvi4* [12]. To determine whether or not *CCS52A1* activity is also responsible for the lethality of *osd1 uvi4*, we crossed a loss-of-function mutation of *CCS52A1* (SALK_083656) with *uvi4/uvi4 osd1/OSD1.* Plants with the *osd1/OSD1 uvi4/uvi4 ccs52a1*/*ccs52a1* genotype were isolated in the F2 population and their progenies were analyzed. Among 96 progenies analyzed, 2 plants were identified as *osd1 uvi4 ccs52a1* homozygous triple mutants, 41 plants as *OSD1/OSD1 uvi4/uvi4 ccs52a1*/*ccs52a1*, and 53 as *osd1/OSD1 uvi4/uvi4 ccs52a1*/*ccs52a1* (Figure 2E). Thus the lethality of female gametophyte and zygote defects of *uvi4 osd1* is partially dependent on the activities of *CCS52A1*. The triple mutant of *osd1 uvi4 ccs52a1* was much smaller than wild-type Col-0 or *ccs52a1* (Figure 2F), suggesting that other *CCS52* genes might mediate the function of *OSD1* and *UVI4* in zygote development.

### The *cpr5* Mutation Largely Suppresses the Endoreduplication Defects in *uvi4* but not the Meiotic Defects in *osd1* Mutant

**Figure 3:**
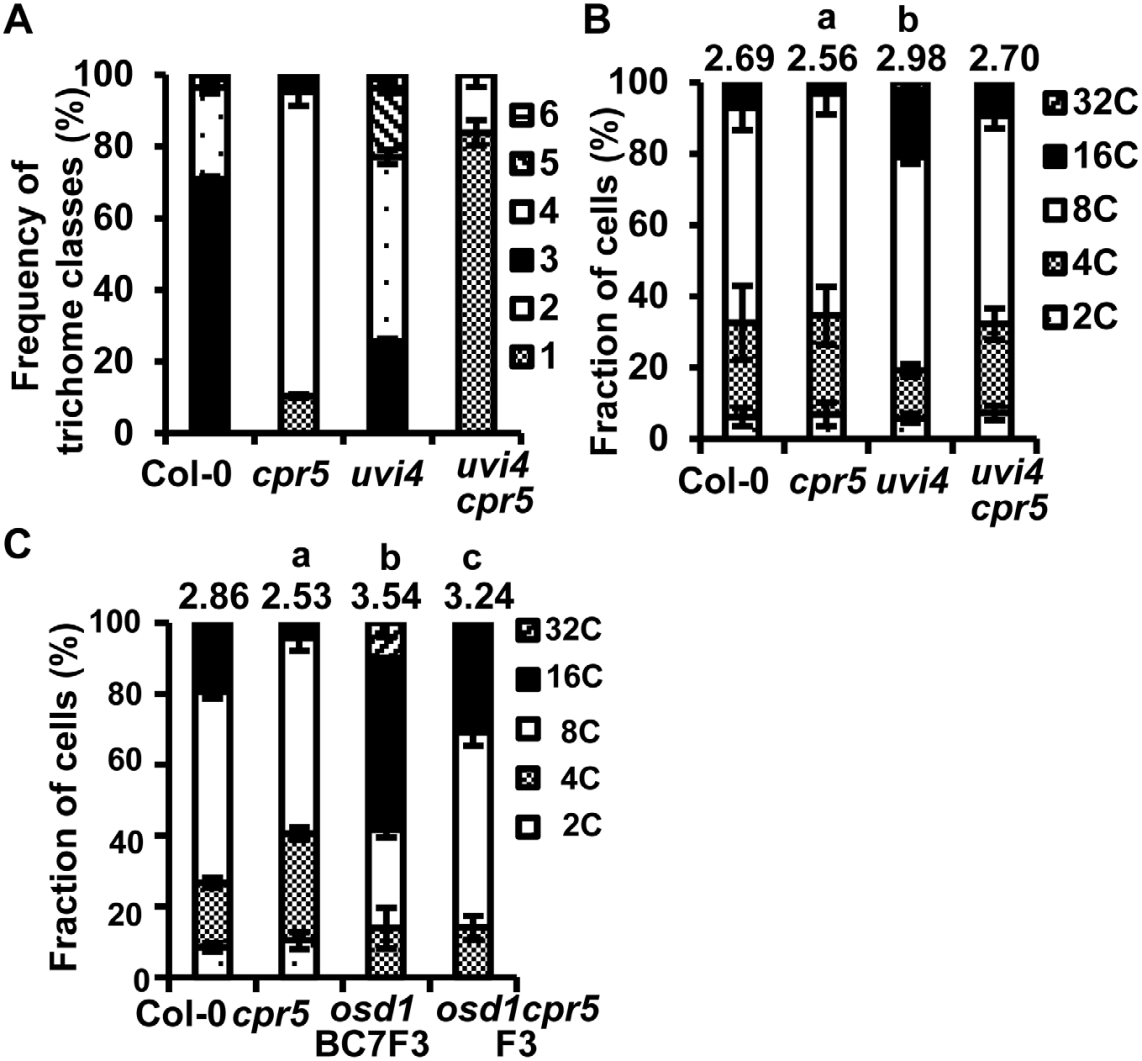
Genetic interactions of *uvi4* and *osd1* with *cpr5* in the regulation of endoreduplication and meiosis. (A) Frequency of cells with different branching numbers in Col-0, *cpr5*, *uvi4*, and *uvi4 cpr5* plants on the forth leaves. Approximately 100 to 150 cells were examined for each genotype. (B) DNA ploidy levels of Col-0, *cpr5*, *uvi4*, and *uvi4 cpr5* shown as percentage of cells with 2C to 32C. (C) DNA ploidy levels of Col-0, *cpr5*, *osd1* BC7F3, and *cpr5 osd1* F3 plants shown as percentage of cells with 2C to 32C. Error bars indicate standard deviations. The number above the column indicates the ploidy index. Numbers in (B) and (C) are averages of three replicas. Ploidy indices were used for statistical analysis, and the letter a indicates a statistical significance determined by student *t*-test. The representative data were shown from two independent measurements.

The fact that overexpression of *OSD1* or *UVI4* confers enhanced disease resistance and reduced endoreduplication prompted us to look at genetic interaction of *OSD1* and *UVI4* with the *CPR5* gene that is also involved in these two processes [22]–[25]. The double l-o-f mutant was generated between *cpr5-2* [22] (referred as *cpr5* from now on) and *uvi4* in Col-0, and analyzed for trichome branching numbers indicative for endoreduplication and ploidy levels [24]. On the adaxial side of the fourth leaf in the three-week-old plants grown under constant light, wild type Col-0 typically had trichomes with three and four branches, the *cpr5* mutant had one or two branches, and *uvi4* had mostly three to five branches (Figure 3A). The *cpr5 uvi4* double mutant had fewer branches than *uvi4* (Figure 3A), indicating that *cpr5* suppressed *uvi4* defects in trichome branching regulation. Intriguingly, the *uiv4 cpr5* double mutant had even fewer trichome branches than *cpr5*. This enhancement of *cpr5* by *uvi4* suggests a complex interaction between *UVI4* and *CPR5* in trichome development. Perhaps the developmental programs are further altered in the *cpr5 uvi4* mutant, which affects the differentiation of trichomes. In parallel, nuclear DNA content in the first pair of leaves was analyzed by flow cytometry. Ploidy indices were calculated as 2.69 in Col-0, 2.56 in *cpr5*, 2.98 in *uvi4* and 2.70 in *uvi4 cpr5* (Figure 3B),. The *cpr5* and *uvi4* mutant had significantly lower and higher ploidy index compared to Col-0, respectively, while the *cpr5 uvi4* double mutant had a similar ploidy index to the wild type Col-0 (Figure 3B), indicating a suppression or compensation of endoreduplication defects of *uvi4* by *cpr5* in leaf cells.

We analyzed the genetic interaction between *osd1* and *cpr5* as well. *OSD1* is essential for the second meiotic division, and loss of function of *OSD1* leads to diploid gametes and tetraploid progenies [14]. The *cpr5* mutant was crossed with the heterozygous *osd1*, and the *cpr5 osd1* double mutant was isolated from the F2 population. Ploidy levels in progenies of these *cpr5 osd1* homozygous F2 plants were measured. As expected, no 2C cells were detected in the progenies of the homozygous *osd1* plants named *osd1* (BC7F3) (Figure 3C), because the genome was duplicated due to the production of diploid gametes. No 2C cells were detected in the *cpr5 osd1* F3 plants either (Figure 3C), indicating that *cpr5* mutation does not suppress the meiotic defect in the *osd1* mutant. Ploidy indices were 2.86 in Col-0, 2.53 in *cpr5*, 3.54 in *osd1* BC7F3 plants and 3.24 in *osd1 cpr5* F3 plants (Figure 3C). The *osd1 cpr5* F3 plants had significantly lower ploidy index than *osd1* BC7F3, similar to the significant reduction of index in *cpr5* compared to Col-0. Thus, a *cpr5* defect exists even in the background of the *osd1* tetraploid.

### The *cpr5* Mutation Partially Suppressed the Lethality of the *uvi4* and *osd1* Double Mutant

**Figure 4:**
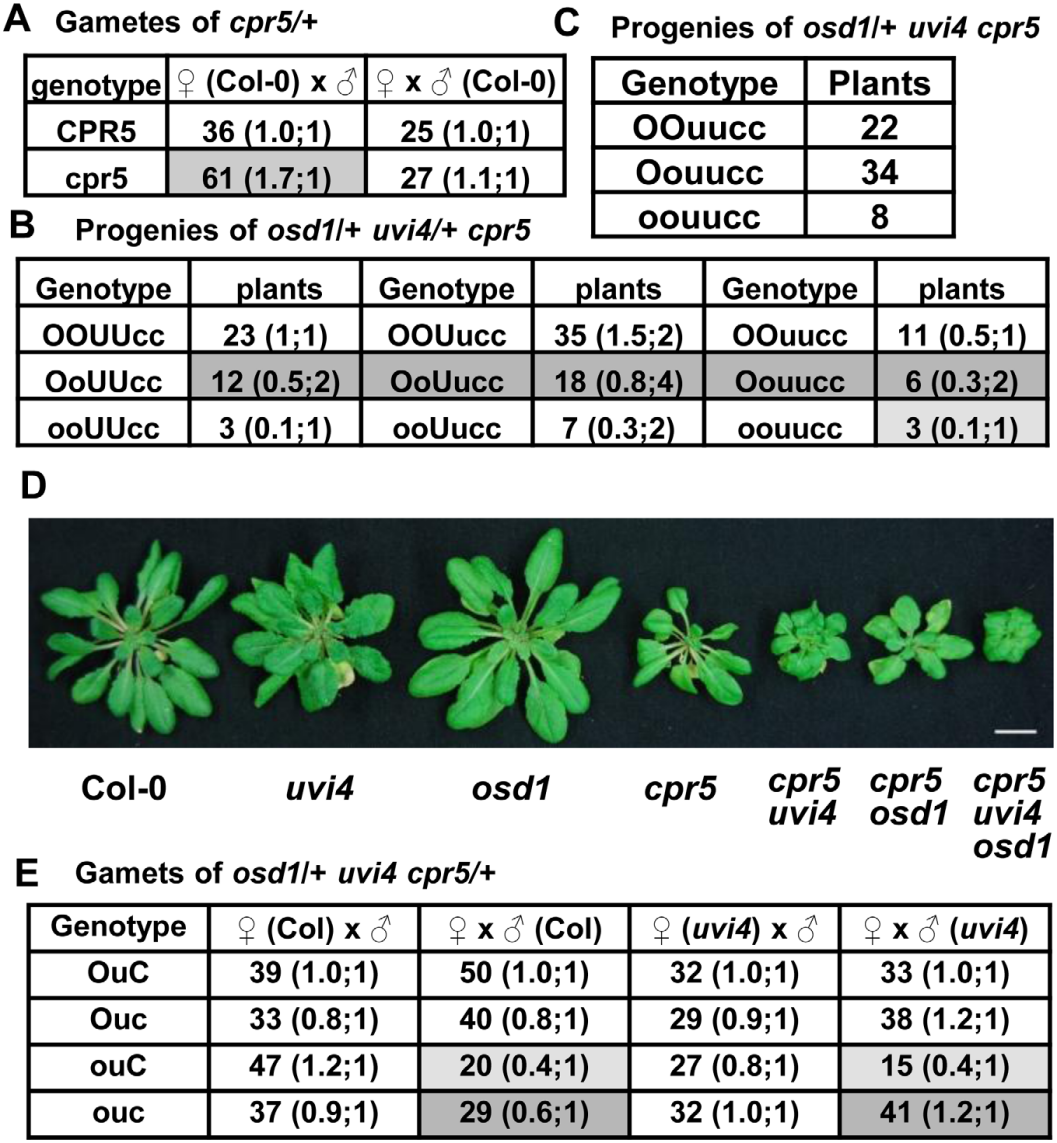
The *cpr5* mutation partially suppresses the lethality of *osd1 uvi4* double mutant. Shown are numbers of plants of each genotype in an analyzed population. The two numbers separated by a semicolon in parentheses are the observed ratios of that genotype relative to the top left genotype (left) and the expected ratios when there is no reduced transmission of the gametes or zygotes (right). (A) Analysis of gamete transmission inferred from of reciprocal crosses between *CPR5/cpr5* and Col-0. The *cpr5* had a higher transmission rate as male gamete (shaded) but not female gamete. The significance was determined by chi-square test (male, P = 0.011<0.05; female, P = 0.78>0.05). (B) Analysis of progenies from *osd1/OSD1 uvi4/UVI4 cpr5/cpr5*. Notice the presence of *osd1/osd1 uvi4/uvi4 cpr5/cpr5* (shaded) while there was no *osd1/osd1 uvi4/uvi4* in progenies of *osd1/OSD1 UVI4/uvi4* (Figure 2A). Also notice that plants with *osd1/OSD1 cpr5/cpr5* genotypes irrespective of the *uvi4* genotypes (darker shaded) were fewer than expected, suggesting a lower transmission of *osd1 cpr5* than *OSD1 cpr5*. The difference between observed segregation ratio and normal segregation ratio was determined by chi-square test (P = 1.07E-14<0.05). (C) Analysis of progenies from *osd1*/*OSD1 uvi4/uvi4 cpr5/cpr5*. (D) Morphology of Col-0, *uvi4*, *osd1*, *cpr5*, *cpr5 uvi4*, *cpr5 osd1*and *cpr5 uvi4 osd1* grown for 6 weeks under 12 h light/12 h dark condition. (E) Analysis of gamete transmission inferred from of reciprocal crosses between *osd1/OSD1 uvi4/uvi4 CPR5/cpr5* and Col-0 or *uvi4*. Notice a lower transmission (20 versus 50 with Col-0 and 15 versus 33 with *uvi4*) of *osd1uvi4CPR5* (shaded) compared to *OSD1uvi4CPR5* as female gametes but an increased transmission rate (29 versus 40 with Col-0 and 41 versus 38 with *uvi4*) of *osd1uvi4cpr5* (darker shaded) compared to that of *osd1uvi4CPR5*. The difference of gamete transmission between *osd1/OSD1 uvi4/uvi4* and *osd1/OSD1 uvi4/uvi4 CPR5/cpr5* was determined by chi-square test (reciprocal cross with Col-0, P = 1.71E-36<0.05; reciprocal cross with *uvi4*, P = 4.20E-37<0.05).

To determine whether or not *cpr5* mutation affects gametophyte development, we first carried out reciprocal test crosses between heterozygous *cpr5/CPR5* and the wild type Col-0 and genotyped their F1 progenies. The female transmission rate of *cpr5* was 108% (27/25) relative to the wild type, and the male transmission rate of *cpr5* was 169% (61/36) relative to the wild type (Figure 4A). This indicates that *cpr5* mutation enhances transmission of male gametophytes.

We subsequently analyzed the interaction between *cpr5* and the *osd1 uvi4* double mutant in gametophyte development. The *osd1/OSD1 cpr5/cpr5* was crossed to *uvi4/uvi4 cpr5/cpr5*, and the *osd1/OSD1 uvi4/UVI4 cpr5/cpr5* plants were selected among the F1 progenies. Analysis of their progenies revealed a reduced transmission of *osd1* gamete relative to the wild-type *OSD*1 gamete in the *cpr5* background, and this reduction did not occur in the wild type background (Figure 2A). In progenies of *osd1/OSD1 uvi4/UVI4 cpr5/cpr5*, plants with the *osd1/OSD1 cpr5/cpr5* genotype relative to those with the *OSD1/OSD1 cpr5/cpr5* genotype were about 50% instead of the expected 200%: 12 versus 23 with *UVI4/UVI4*, 18 versus 35 with *uvi4/UVI4*, and 6 versus 11 with *uvi4/uvi4* (Figure 4B). This indicates that the *osd1 cpr5* gamete has a lower transmission rate than *OSD1 cpr5.* It is likely that *osd1* has a lower transmission rate but goes undetected as its transmission activity nevertheless reaches a threshold. But this activity becomes lower than threshold when *cpr5* confers a higher threshold.

Significantly, we identified *osd1/osd1 uvi4/uvi4 cpr5/cpr5* plants (3 out of 118) from the progenies of *osd1/OSD1 uvi4/UVI4 cpr5/cpr5* (Figure 4B). In addition, when progenies of *osd1/OSD1 uvi4/uvi4 cpr5/cpr5* were analyzed, 8 out of 64 plants were genotyped as *osd1/osd1 uvi4/uvi4 cpr5/cpr5* (Figure 4C). Therefore, *cpr5* rescued the embryo lethal defect of *osd1/osd1 uvi4/uvi4* zygotes and likely increased the survival and transmission of female gametophytes of *osd1 uvi4*. While viable triple mutant of *osd1 uvi4 cpr5* was obtained, these plants had more compact rosette leaves and were much smaller than *cpr5* (Figure 4D). We further determined the gamete transmission rates by crossing *osd1/OSD1 uvi4/uvi4 cpr5/CPR5* to Col-0 and *uvi4* respectively. Progeny genotyping show that the transmission rate of *osd1 uvi4* female gametophytes was about 40% (20/50) and 45% (15/33) of that of *OSD1 uvi4* when crossed to Col-0 and *uvi4* respectively. The transmission rate of *osd1 uvi4 cpr5* relative to that of *OSD1 uvi4 cpr5* was increased to 73% (29/40) and 108% (41/38) in crosses to Col-0 and *uvi4* respectively (Figure 4E). Thus, *cpr5* suppressed the lethality in the female gamete of *osd1 uvi4*. The difference of apparent rescue extent of the female gametophyte in crosses to the wild type and *uvi4* might result from a different survival rate at the zygote stage.

### The *cpr5* Mutation Activates the Expression of Cell Cycle Genes

**Figure 5:**
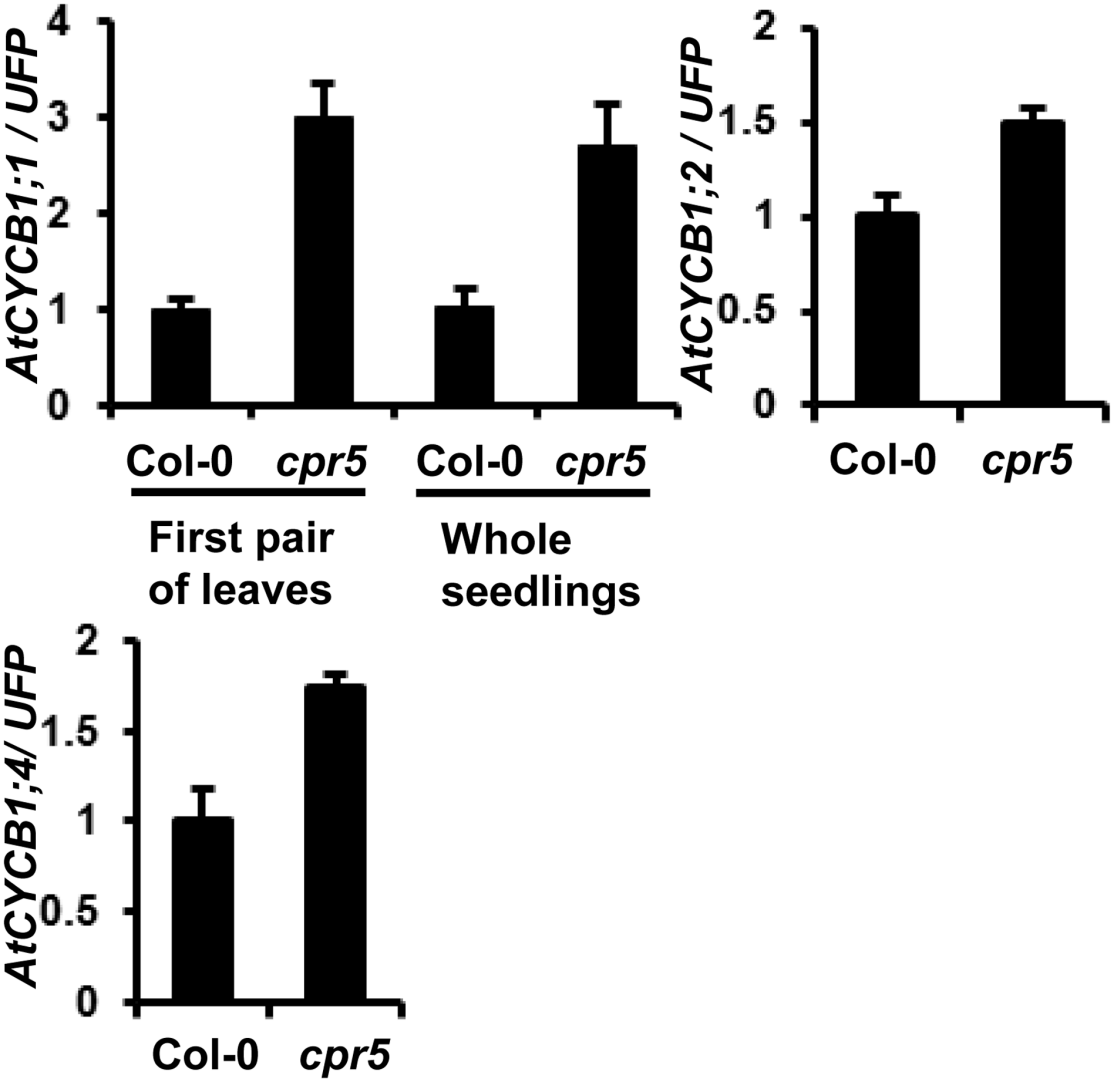
Expression of cell cycle genes in *cpr5* mutant. Expression levels of *AtCYCB1;1* in both the first pair of leaves and whole seedlings of 2-week-old plants, and *AtCYCB1;2* and *AtCYCB1;4* in whole seedlings analyzed by quantitative real time RT-PCR. Error bars indicate standard deviations.

Endoreduplication is inhibited by the loss of *CPR5* function. We further investigated how cell cycle progression is affected in *cpr5* by analyzing expression of genes specific to distinct phase of the cell cycle. These include G1-phase *CYCD3;3*, S-phase histone *H3.1*, G2-phase *CYCA2;1* and G2 to M transition *CYCB1;1* [37], [39]. *CYCB1;1*, but not the other two genes, was found to have an altered expression in *cpr5* compared to wild-type Col-0 by RT-PCR. The *CYCB1;1* expression was two times more in *cpr5* than in the wild type when the first pair of leaves or the whole seedling were sampled (Figure 5, S2). We subsequently looked at expression of other members of the *CYCB1* family by qRT-PCR and found that the expression levels of *CYCB1;2* and *CYCB1;4* in *cpr5* were 1.5 and 1.7-fold respectively of that in Col-0 (Figure 5). These data indicate that *cpr5* affects the expression of B1-type cyclin genes which might be involved in endoreduplication regulation [40].

### Both *uvi4* and *osd1* Mutations Enhance the Disease Resistance of *cpr5*

**Figure 6:**
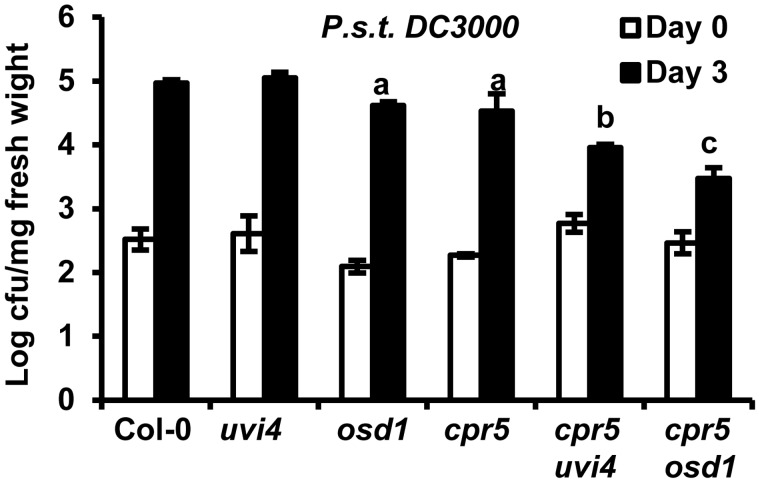
Both *uvi4* and *osd1* mutations enhance defense responses in *cpr5*. Bacterial growth assay in Col-0, *uvi4*, *osd1*, *cpr5*, *cpr5 uvi4* and *cpr5 osd1* (all diploid plants) inoculated by spray inoculation of *Pst* DC3000. Error bars indicate standard deviations. Letters a, b and c indicate the statistical significance determined by student *t*-test.

To determine how the *CPR5* might interact with *UVI4* and *OSD1* to affect defense responses, we analyzed diseases resistance phenotypes of *cpr5* mutant combinations with *osd1* or *uvi4*. The growth of virulent bacterial pathogen *Pseudomonas syringae* pv. *tomato DC3000* (*Pst* DC3000) were analyzed in *cpr5 uvi4* and *cpr5 osd1* double mutants. To avoid genome duplication effects from the *osd1* mutation, we isolated *cpr5 osd1* from progenies of *cpr5/cpr5 OSD1/osd1* plants. Four-week old plants were spray inoculated with the bacterial pathogen. At day 3 after inoculation, both the *cpr5* and *osd1* single both supported 3 times less pathogen growth than the wild type, while *uvi4* supported the same amount of growth as the wild type (Figure 6). The double mutants *cpr5 uvi4* and *cpr5 osd1* supported even less growth than the *cpr5* single mutant, with a 4-fold and 10-fold reduction compared to *cpr5*, respectively (Figure 6). This indicates that the *cpr5* mutation has a synergistic effect on disease resistance with the *uvi4* and *osd1* mutations.

## Discussion

**Figure 7:**
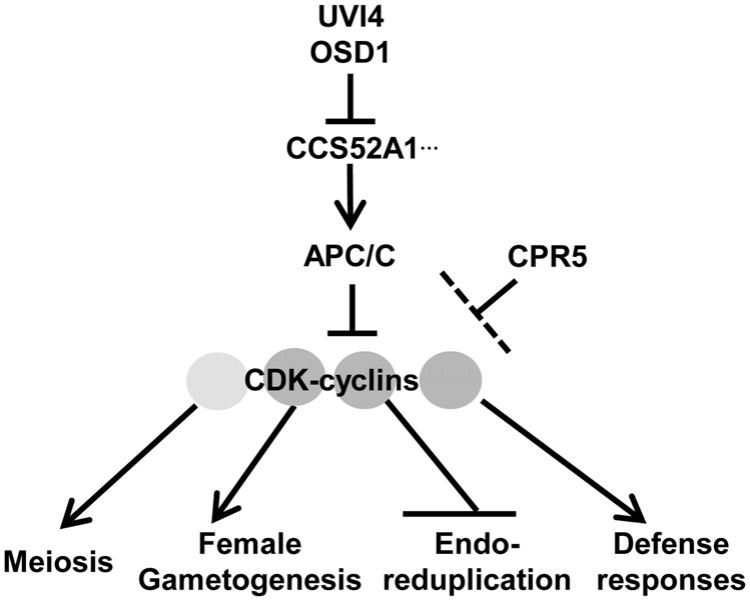
Roles of the *OSD1*, *UVI4* and *CPR5* genes. Both UVI4 and OSD1 negatively regulate APC/C activities partially through their interaction with CCS52A1. APC/C inhibits activities of various CDK-cyclin complexes (indicated by shaded circles) each of which regulates processes including meiosis, female gametogenesis, endoreduplication and defense response. CPR5 negatively regulates the transcript levels of some cyclin genes and thus the activities some CDK-cyclin complexes (indicated by darker shades), and affects female gametogenesis, endoreduplication and defense responses. The exact action point of CPR5 is yet to be determined.

Previous studies on the loss of function mutants show that *OSD1* and *UVI4* inhibit APC/C activity and thus regulate cell cycle progression in meiosis, endoreduplication, and gametophyte development. Through analyzing their overexpression phenotypes, we further demonstrate that the level of *OSD1* and *UVI4* is a critical determinant for cells to enter regular mitosis or endoreduplication. This study also establishes that *CCS52A1* mediates the overlapping function of *OSD1* and *UVI4* in gametophyte development. OSD1 and UVI4 were postulated to inhibit members of the CCS52 protein family, but the particular member that they inhibit is not clear in each of the diverse processes they regulate. The loss of *ccs52a1* mutation was previously shown to completely rescue the *uvi4* defect in endoreduplication [12]. Here we found that the *ccs52a1* mutation partially rescued the lethality of *osd1 uvi4* (Figure 2). This partial suppression may be due to the functional redundancy of *CCS52A1* with two other homologs, *CCS52A2* and *CCS52B*. Both *CCS52A1* and *CCS52A2* control the onset and progression of endoreduplication [8], [10], and overexpression of *CCS52B* enhances the endoreduplication defects in *uvi4* [13]. Thus, loss of both *OSD1* and *UVI4* function could release the inhibition on multiple CCS52 proteins (Figure 7).

Furthermore, this study reveals *CPR5* as an additional player in cell cycle regulation and this function largely antagonizes that of *UVI4* and *OSD1* (Figure 7). The *cpr5* mutation suppressed multiple defects in *uvi4* single and *uvi4 osd1* double mutants, including the endoreduplication phenotype of *uvi4* and lethality of *osd1 uvi4* double mutant (Figure 3, 4). Therefore, *CPR5* functions either in parallel to or downstream of *UVI4* and the function of *CPR5* and *UVI4* are largely opposite. However, the interaction between *CPR5* and *UVI4* is complex. The *cpr5* defect in trichome branching was enhanced by *uvi4* although the *uvi4* single mutant had an opposite phenotype to *cpr5* (Figure 3A). The enhancement of *cpr5* phenotype by *uvi4* was also seen in the disease resistance (Figure 6B). This unusual interaction might be due to an overlapping function between *OSD1* and *UVI4*. The loss of *UVI4* might be compensated by *OSD1* in a tissue and developmental stage dependent manner. For instance, the *cpr5* phenotype can be enhanced by *uvi4* due to the higher expression of *OSD1* in trichome cells but not necessarily other cells in the leaf.

The converging point of regulation by *CPR5* and *UVI4* might be on the cyclins. *OSD1* and *UVI4* are shown to regulate cyclins mainly at the protein level through the E3 ligase complex APC/C [12], [13], and a transcriptional regulation of *CYCB1;1* by APC/C also takes place [41]. *CPR5* directly or indirectly affects transcript level of cyclins although the mechanism is unknown. An upregulation of *CYCB1;1*, *CYCB1;2*, and *CYCB1;4* is observed in the leaves of the *cpr5* mutant, which may account for the reduced endoreduplication in mutant leaves. The *cpr5* mutation itself promoted male gametophyte transmission (Figure 4A). Upregulation of *CYCB1;1* or other cyclins might occur during gametophyte development in *cpr5* leading to accelerated development and higher transmission of male gametophytes. Therefore, the *cpr5* mutation may compensate the lower amount of cyclin proteins in the *uvi4* mutant by upregulating the transcript of cyclins and thus suppresses the uvi4 defects (Figure 7). It is yet to determine whether or not the cyclin genes are the converging regulatory nodes of *OSD1*/*UVI4* and *CPR5* and, if so, what cyclins they are.

This study further supports a connection between defense responses and cell cycle progression. *CPR5*, *UVI4*, and *OSD1* are all implicated in cell cycle regulation as well as defense response regulation. It is possible that cell cycles are often manipulated by pathogens. It has been observed that powdery mildew infection triggered endoreduplication at the infection site [42]. Recent study reported that an APC/C component, APC8, is one of the 5 significant hubs targeted by pathogen effectors [43]. Therefore, cell cycle machinery and consequently cell cycle progression are likely manipulated by various bacterial and oomycete pathogens. This manipulation, without counteracting effects from plants, could be beneficial to pathogen. However, these manipulations by pathogens might become ‘guarded’ by plant R proteins to trigger defense responses. No R proteins are known to interact with APC8 [43], however expression of *R* genes could be affected by cell cycle progression. For instance, the *R* gene *SNC1* has an increased transcript level in mutants of several APC components including *APC8*, *APC13* and *APC10* as well as overexpression of *OSD1* [20]. This change of *R* gene expression is dependent on cyclins as *SNC1* upregulation in the *OSD1* overexpression line is abolished by the *cycb1;1* mutation [20]. The *cpr5* has a higher expression of *CYCB1;1* and *CYCB1;2* which potentially can lead to upregulation of *R* genes as well. Therefore, upregulation of *R* gene transcript might be a new mechanism in monitoring effectors in addition to the up-regulation of R proteins. Cell cycle has been shown to affect gene expression [44]–[46] and the expression of certain *R* genes might be susceptible to perturbation of cell cycle and therefore form the basis of pathogen recognition.

In sum, we uncovered a role of *CPR5* that antagonizes with that of *UVI4* and *OSD1* in cell cycle regulation (Figure 7). Both UVI4 and OSD1 inhibit the activity of APC/C through the interaction with APC/C activator CCS52 proteins. APC/C degrades cyclins and inhibits activities of CDK-cyclin complex which are critical for the female gametophyte development, trichome branching, and plant defense responses. *CPR5* may negatively regulate cell cycle components such as cyclins at the transcript level. The critical role of immune regulator *CPR5* in cell cycle regulation further supports a tight connection between defense responses and the regulation of cell cycle progression.

## Supporting Information

Figure S1 **Female gametophyte development of** ***osd1uvi4*** **.** (A) Confocal laser scanning microscopy images of the terminal female gametophyte in an *osd1/OSD1 uvi4/uvi4* pistil which contained wild-type female gametophyte at FG7 (left panel) and abnormal female gametophyte arrested at FG1 (right panel). SEN, secondary endosperm nucleus; CV, central vacuole; EN, egg nucleus; SN, synergid nucleus; N, nucleus; V, vacuole. (B) Confocal laser scanning microscopy of female gametophytes at early developmental stages in the *osd1/+ uvi4* pistil. All gametes showed either wild-type FG1 (left panel) or FG2 (right panel) features at this stage. FM, functional megaspore. N, uninucleate. DM, degenerating megaspore. MN, micropylar nucleus. CN, chalazal nucleus. Scale bar = 10 µm.
(TIF)

Figure S2 **Gene expression of cell cycle marker genes in** ***cpr5*** **mutant.** Analysis of cell cycle marker genes in the first pair of leaves and the whole seedlings of two-week old plants by RT-PCR. *AtGAPC1* was used as a control.
(TIF)

Table S1 **List of primers for qRT-PCR analysis.**
(DOCX)
