# Supplementary figures and images for: Interaction of CPR5 with Cell Cycle Regulators UVI4 and OSD1 in Arabidopsis
Source: PLoS One. 2014 Jun 19;9(6):e100347. doi: 10.1371/journal.pone.0100347 (PMC4063785; doi:10.1371/journal.pone.0100347)

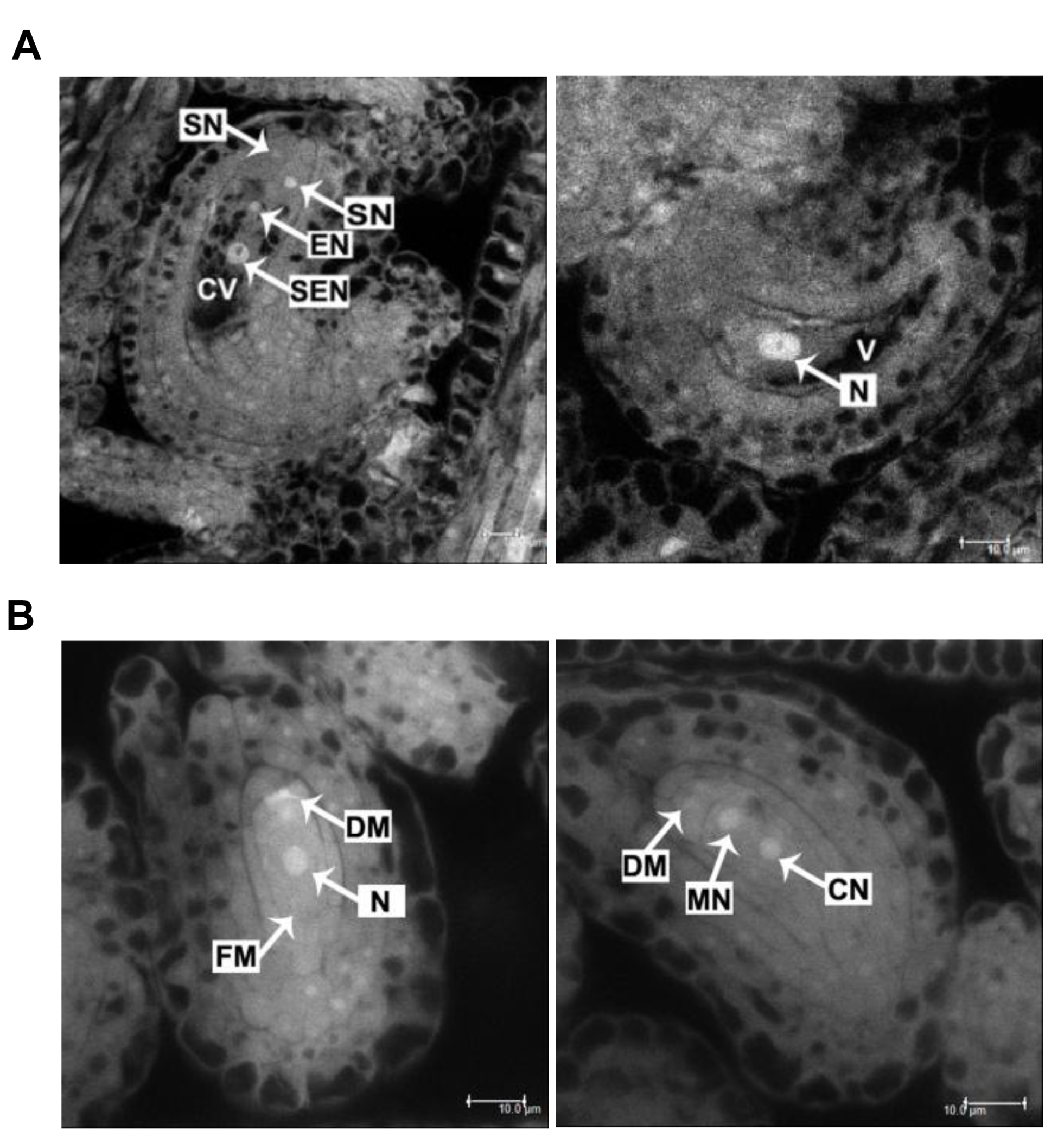

Supplement: Figure S1 — Female gametophyte development of osd1uvi4 . (A) Confocal laser scanning microscopy images of the terminal female gametophyte in an osd1/OSD1 uvi4/uvi4 pistil which contained wild-type female gametophyte at FG7 (left panel) and abnormal female gametophyte arrested at FG1 (right panel). SEN, secondary endosperm nucleus; CV, central vacuole; EN, egg nucleus; SN, synergid nucleus; N, nucleus; V, vacuole. (B) Confocal laser scanning microscopy of female gametophytes at early developmental stages in the osd1/+ uvi4 pistil. All gametes showed either wild-type FG1 (left panel) or FG2 (right panel) features at this stage. FM, functional megaspore. N, uninucleate. DM, degenerating megaspore. MN, micropylar nucleus. CN, chalazal nucleus. Scale bar = 10 µm. (TIF) [file pone.0100347.s001.tif]

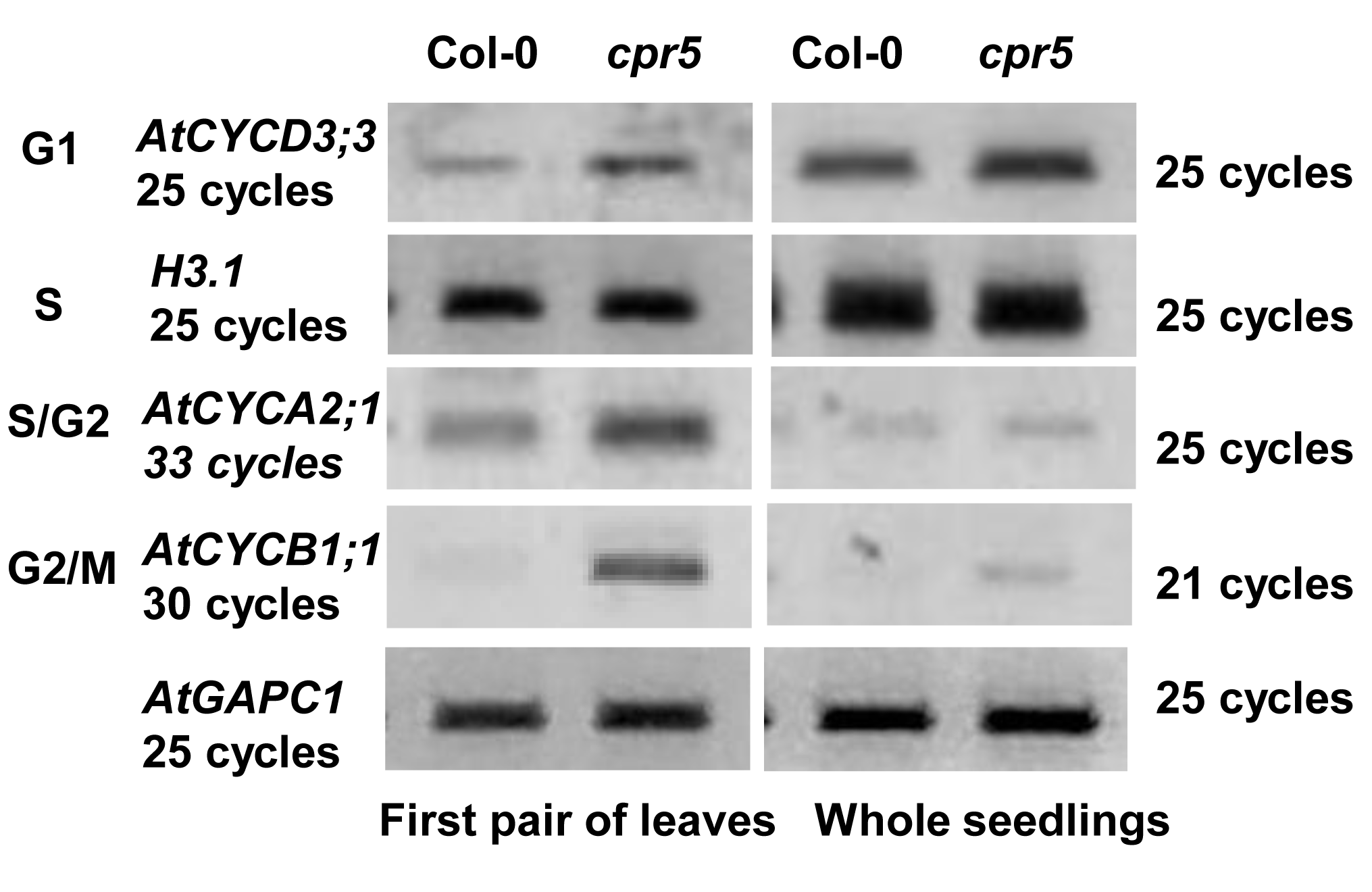

Supplement: Figure S2 — Gene expression of cell cycle marker genes in cpr5 mutant. Analysis of cell cycle marker genes in the first pair of leaves and the whole seedlings of two-week old plants by RT-PCR. AtGAPC1 was used as a control. (TIF) [file pone.0100347.s002.tif]
