# Supplementary material for: Interaction of CPR5 with Cell Cycle Regulators UVI4 and OSD1 in Arabidopsis
Source: PLoS One. 2014 Jun 19;9(6):e100347. doi: 10.1371/journal.pone.0100347 (PMC4063785; doi:10.1371/journal.pone.0100347)
Supplement: Table S1 — List of primers for qRT-PCR analysis. (DOCX) [file pone.0100347.s003.docx]

**Table S1.** List of primers for qRT-PCR analysis

| Gene Name | Primer Name | Primer Sequences | References |
| --- | --- | --- | --- |
| UFP | UFP-rF: | CCAgCAgACATggAggTTTTgggg | Zhu, Y.,et al., (2011). *Plant J*, 66, 443-455. |
|  | UFP-rR: | TgTTgTCTgTCATTTCTTggCCAgT |  |
|  |  |  |  |
| CYCB1;1 | CYCB1;1-qRT-F: | TAAGCAGATTCAGTTCCGGTCAAC | Iwata, E., et al., (2011). *The Plant cell*, 23, 4382-4393. |
|  | CYCB1;1-qRT-R: | GGGAGCTTTACGAAAGAAATACTCC |  |
|  |  |  |  |
| CYCB1;2 | CYCB1;2-qRT-F: | TTTGAGCAGTCCATAATCTCAGAC | Zhu, Y.,et al., (2011). *Plant J*, 66, 443-455. |
|  | CYCB1;2-qRT-R: | ATTACGACACCTTGACGTTCTGTC |  |
|  |  |  |  |
| CYCB1;4 | CYCB1;4-qRT-F: | TAAGAGCTCGAAGCAAGGCTG | this study |
|  | CYCB1;4-qRT-R: | CGTATTCCACAGCAGCCAGT |  |
